# Detection of 1014F *kdr *mutation in four major Anopheline malaria vectors in Indonesia

**DOI:** 10.1186/1475-2875-9-315

**Published:** 2010-11-08

**Authors:** Din Syafruddin, Anggi PN Hidayati, Puji BS Asih, William A Hawley, Supratman Sukowati, Neil F Lobo

**Affiliations:** 1 Eijkman Institute for Molecular Biology, Jalan Diponegoro, 69, Jakarta 10430, Indonesia; 2 Department of Parasitology, Hasanuddin University, Makassar 90245, Indonesia; 3 Division of Malaria and Parasitic Diseases, Centers for Diseases Control and Prevention, Atlanta, USA; 4 Health Ecology Research & Development Centre, National Institute of Health, Research and Development, Jalan Percetakan Negara 29, Jakarta, Indonesia; 5 Eck Institute for Global Health, University of Notre Dame, Notre Dame, Indiana, USA

## Abstract

**Background:**

Malaria is a serious public health problem in Indonesia, particularly in areas outside Java and Bali. The spread of resistance to the currently available anti-malarial drugs or insecticides used for mosquito control would cause an increase in malaria transmission. To better understand patterns of transmission and resistance in Indonesia, an integrated mosquito survey was conducted in three areas with different malaria endemicities, Purworejo in Central Java, South Lampung District in Sumatera and South Halmahera District in North Mollucca.

**Methods:**

Mosquitoes were collected from the three areas through indoor and outdoor human landing catches (HLC) and indoor restinging catches. Specimens were identified morphologically by species and kept individually in 1.5 ml Eppendorf microtube. A fragment of the *VGSC *gene from 95 mosquito samples was sequenced and *kdr *allelic variation determined.

**Results:**

The molecular analysis of these anopheline mosquitoes revealed the existence of the 1014F allele in 4 major malaria vectors from South Lampung. These species include, *Anopheles sundaicus, Anopheles aconitus, Anopheles subpictus *and *Anopheles vagus*. The 1014F allele was not found in the other areas.

**Conclusion:**

The finding documents the presence of this mutant allele in Indonesia, and implies that selection pressure on the *Anopheles *population in this area has occurred. Further studies to determine the impact of the resistance allele on the efficacy of pyrethroids in control programmes are needed.

## Background

The archipelago nation of Indonesia is commonly divided into two regions based on malaria endemicity; Java and Bali, inhabited by approximately 62% of the total Indonesian population, is classified as hypo-endemic area, whereas the more sparsely populated outer islands, including Sumatera, Kalimantan, Sulawesi, Nusa Tenggara, Maluku and Papua, have malaria at much higher levels, ranging from hypo to hyper endemic. All four species of human malaria are found in Indonesia. Formerly, *Plasmodium malariae *and *Plasmodium ovale *were mostly found in the eastern part of Indonesia - Nusa Tenggara Timur and Papua - but in recent years, *P. malariae *has been detected in western parts of the archipelago as well [1,2]. Vivax malaria was more predominant in Java while in the outer islands the prevalence of vivax and falciparum malaria have been equivalent [2].

During the pre-eradication era, malaria morbidity country-wide was as higher than it is in eastern Indonesia at present. The estimated annual malaria cases and deaths were around 30 million and 120,000, respectively [2]. Recently, the annual parasite incidence (API) varied substantially among the provinces in Java and Bali, but in the year of 2007, the highest API was detected in Bali province. In areas outside Java and Bali, the highest annual malaria incidence (AMI) was detected in West Papua Province [3].

Malaria parasites in Indonesia are transmitted by more than a dozen species of *Anopheles *mosquitoes that vary markedly in biological attributes, including patterns of blood feeding, response to insecticides, resting behavior and larval habitats. This variation impacts the effectiveness of interventions such as insecticide-treated nets (ITNs), indoor residual spraying (IRS) and larval habitat treatments or modifications [4-9]. Resistance to insecticides may also compromise the effectiveness of interventions. Determining the impact of resistance on malaria transmission is complicated by the multiple molecular resistance mechanisms and by the dynamics of the emergence and spread of resistance genotypes in vector populations [8,9].

Insecticide resistance mechanisms may have varying impact on the effectiveness of insecticide-based control programmes. Knowledge of resistance mechanisms is necessary to guide insecticide use in vector control programmes. Molecular studies over the past decades have identified several polymorphisms associated with the resistance phenotype; eg. resistance against pyrethroids and DDT, known as knock-down resistance (*kdr*), has been linked to mutations in the para-type, voltage-gated sodium channel (*VGSC*) gene. Knockdown resistance has also been described in several insect species [10-15]. In the African malaria vector, *Anopheles gambiae *s.s, two substitutions at codon 1014 (L1014F and L1014S) of domain II of the sodium channel gene have been associated with knockdown resistance [10,16-19].

A nation-wide malaria control programme was launched by the government of Indonesia in 1952 [20]. This effort included case treatment with chloroquine and vector control using insecticide residual spraying (IRS) with dichloro-diphenyl-trichloro-ethane (DDT). Following the retraction of DDT in 1970s, organophosphates, carbamates and synthetic pyrethroids were introduced to replace DDT. Some insecticides have also concomitantly been used for controlling agricultural pests [21].

Resistance of malaria vectors to various insecticides in Indonesia has been documented. DDT and dieldrin resistance has been reported in *Anopheles aconitus *- an important vector in rural Java and Bali [22-26]. Resistance to DDT was also reported in *Anopheles sundaicus*, the main malaria vector in the coastal area of East Java, Lampung and Cilacap [24]. Biochemical assays to detect sensitivity of *Anopheles maculatus, An. aconitus *and *An. sundaicus *to organophosphates and carbamates reported tolerance or resistance ranging from 2.9% to 33.3%. Resistance to pyrethroids has also been reported in *Anopheles spp. *in Jepara, Central Java [21]. The present study aims to explore the allelic distribution of *VGSC *gene mutations among malaria vectors from 3 malaria endemic areas in Indonesia.

## Methods

### Study areas

**Figure 1:**
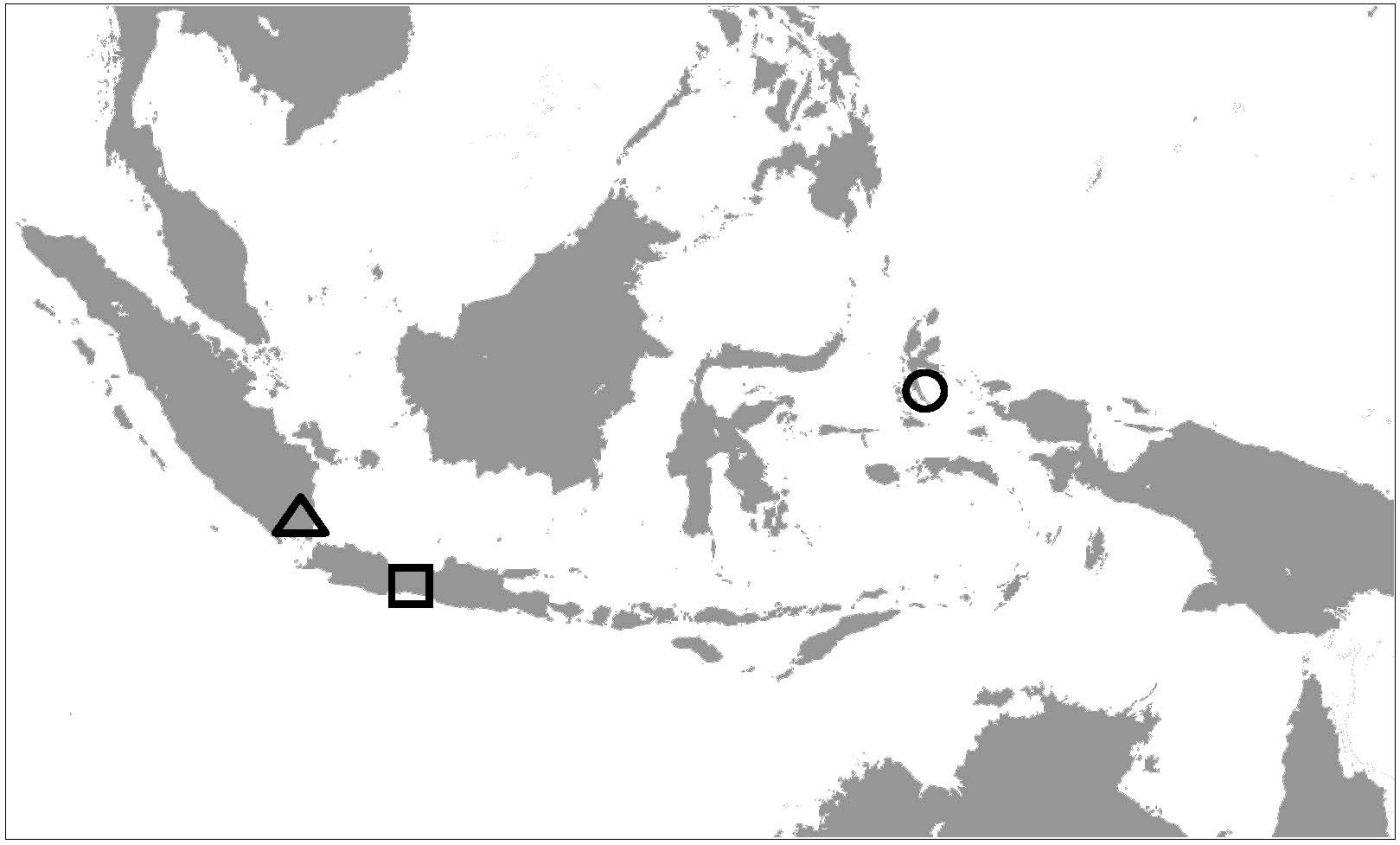
**The geographic locations of study sites in the map of Indonesia (square boxes) **. *Anopheles spp. *were collected from the three areas of Indonesia representing different malaria endemicities, (□) = Purworejo (hypo-endemic), (Δ) = South Lampung (meso-endemic), and (ο) = South Halmahera (hyper-endemic). Drawn not to scale.

Female anopheline mosquitoes were collected from three parts of Indonesia with different malaria endemicities, Purworejo in the central Java Province (hypo-endemic), South Lampung District in South Lampung Province (meso-endemic), the Southern tip of Sumatera, and South Halmahera District in North Mollucca Province (hyper-endemic) (Figure 1). The mosquitoes were collected by indoor and outdoor human landing catchs and resting catches during the period of November 2008-October 2009. Mosquitoes were morphologically identified to species using a key for female anopheles in Indonesia [26], desiccated individually in 1.5 ml Eppendorf microtubes with silica gel and stored at 4°C until analysis.

### Extraction of mosquito DNA

Mosquitoes were ground with teflon pestles in 1.5 ml Eppendorf microtubes containing 25 μl of grinding buffer (0.1 M NaCl, 0.2 M sucrose, 0.1 M Tris HCl, 0.05 M EDTA, and 0.5% sodium dodecyl sulfate (SDS); (pH adjusted to 9.2). Mosquito DNA was extracted according to a procedure described previously [27]. DNA was either used immediately for a polymerase chain reaction (PCR) or stored at -20°C for later analysis.

### Gene amplification with PCR

A portion of the *kdr *gene was amplified using seminested-PCR employing oligos that have been previously published: AgF_*kdr *(5' GAC CAT GAT CTG CCA AGA TGG AAT 3') and AgR_*kdr *(5' GCA AGG CTA AGA AAA GGT TAA GCA 3') with slight modification [28]. The modification included the use of an external reverse oligo, An.*kdr*_R2 (5' GAG GAT GAA CCG AAA TTG GAC 3') as the single-step PCR using the previously published *An. gambiae *based oligos commonly failed to yield amplicons in these Indonesian species. Cycling condition for 1^st ^PCR using oligos AgF_*kdr *x An.*kdr*_R2 was denaturation at 94°C for 5 min, annealing at 45°C for 30 sec, extension at 72°C for 1 min 30 sec (1 cycle) and denaturation at 94°C for 30 sec, annealing at 50°C for 30 sec, extension at 72°C for 1 min (29 cycles); and for 2^nd ^PCR using AgF_*kdr *x An.R_*kdr *with denaturation at 94°C for 5 min, annealing at 45°C for 30 sec, extension at 72°C for 1 min (1 cycle) and denaturation at 94°C for 30 sec, annealing at 50°C for 30 sec, extension at 72°C for 40 sec (39 cycles). All reactions were carried out in 50 μl reaction mixtures containing 50 mM KCl, 10 mM Tris-HCl pH 8.3, 1.5 mM MgCl_2_, 200 mM dNTP, 1 unit of Tag polymerase, and a pair of primers (20 pM each). One to five microliters of DNA was used as template in the first reaction and 1-2 μl of the first round PCR was used as template in the second round of PCR. The PCR products of approximately 260 bp in size were sequenced in all individuals.

## Results

### Mosquito collection

In Lampung, 10 species of anopheles were caught with *An. sundaicus *being the dominant species (Table 1). In Purworejo, eight anopheles species were collected and *Anopheles vagus *was the dominant species. In South Halmahera, five Anopheles species were collected with *An. vagus *and *Anopheles kochi *being relatively dominant. All mosquitoes were collected with indoor and outdoor human landing catches or indoor resting catches.

**Table 1:** List of Anopheles species collected at each study site and methods of collection

			Collection Method			
			
Study sites	Species	Number of mosquitoes collected	Human Landing Catch (HLC)	Resting	Outdoor Landing	Indoor Landing
South	*An. sundaicus*	591	483	108	-	-
Lampung	*An. vagus*	11	9	2	-	-
	*An. subpictus*	2	1	1	-	-
	*An. indefinitus*	1	-	1	-	-
	*An. tessellatus*	8	7	1	-	-
	*An. aconitus*	7	7	-	-	-
	*An. minimus*	2	2	-	-	-
	*An. kochi*	2	2	-	-	-
	*An. annularis*	4	2	2	-	-
	*An. barbirostris*	1	1	-	-	-
						
Purworejo	*An. aconitus*	31	-	16	11	4
	*An. barbirostris*	17	-	12	3	2
	*An. vagus*	117	-	83	33	1
	*An. flavirostris*	15	-	3	7	5
	*An. annularis*	2	-	2	-	-
	*An. balabanceensis*	10	-	-	8	2
	*An. tessellatus*	1	-	-	-	1
	*An. maculatus*	2	-	-	2	-
						
Halmahera	*An. vagus*	15	1	13	1	-
	*An. kochi*	8	-	8	-	-
	*An. punctulatus*	1	-	1	-	-
	*An. barbumrosus*	1	1	-	-	-
	*An. farauti*	3	-	-	3	-

### PCR amplification and DNA sequencing of the fragment of *VGSC *gene of various anopheles species from Indonesia

**Figure 2:**
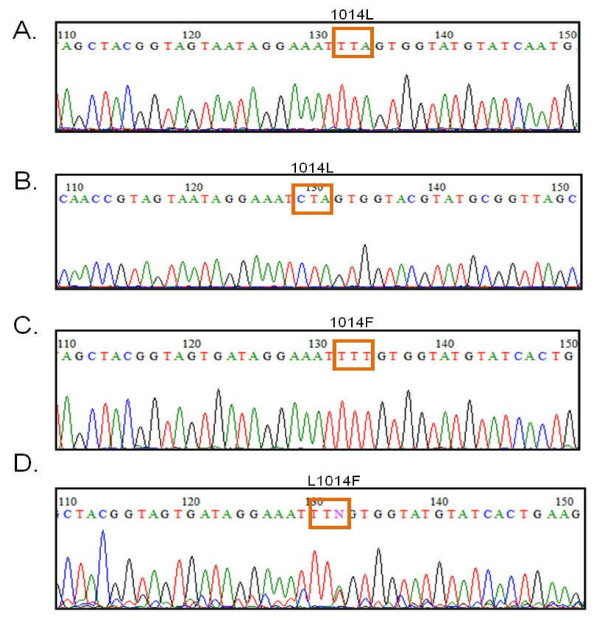
**Electropherogram of the DNA sequencing of *VGSC *gene fragment from *Anopheles tesselatus *(A), *Anopheles balabacensis *(B), *Anopheles sundaicus *(C and D) from Indonesia **. A and B indicate the wildtype allele, 1014L (TTA and CTA). C indicates resistance allele 1014F (TTT), and D indicates the mixed allelic types between L/F.

**Figure 3:**
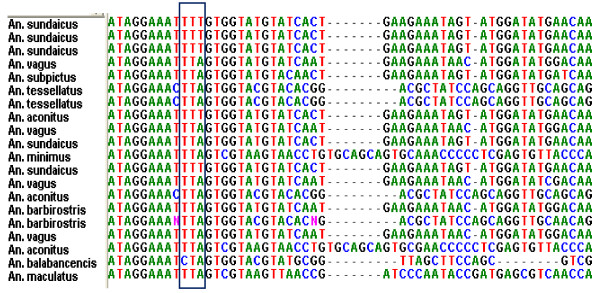
**DNA sequence aligment of the fragment of *VGSC *gene encompassing nucleotides corresponding to the codon 1014 in various anopheles species from Indonesia **. The *kdr-w *allele (TTT) is found either in homozygous- or in heterozygous form.

Using primers that were designed based on the published sequence of *An. gambiae VGSC *gene [GenBank Acc no. AY615628, AY533850, AYDQ026447], amplicons were obtained from 16 *Anopheles spp *collected from Lampung, Purworejo and South Halmahera, respectively. The amplicons of approximately 260 bp in size, were then prepared for DNA sequencing. The alignment of DNA sequencing results of each species is shown in Figure 2. As expected, the DNA sequence of the *VGSC *gene varied significantly among the *Anopheles *species analyzed, but the deduced amino acid sequences indicated a high level of sequence conservation (Figure 3).

### Distribution of kdr gene polymorphisms

Analysis of DNA sequences of 95 amplicons representing 16 species of *Anopheles *species indicated that the 1014F polymorphism of the *VGSC *gene, popularly known as 1014F allele was detected in *An. sundaicus, An. aconitus, Anopheles subpictus *and *An. vagus *isolated from South Lampung District, Lampung Province in Indonesia. This allele was found in either homozygous or heterozygous form. The 1014S allele was not found in any of the *Anopheles *species examined. The allele frequency of *kdr *alleles in each species is shown in Table 2. The overall allele frequency of 1014L and 1014F alleles among the all species of the anopheles examined in Lampung was 44.2% and 55.8%, respectively.

**Table 2:** Frequency of *kdr *allele in each *Anopheles *species examined at each study site

			Genotype frequency (%)			Allele Frequency (%)	
			
Study sites	Species	Total of samples analysed	L/L	L/F	F/F	L	F
South	*An. sundaicus*	40	22.5	10	67.5	27.5	72.5
Lampung	*An. vagus*	5	80	20	0	70	30
	*An. subpictus*	2	50	0	50	50	50
	*An. indefinitus*	1	100	0	0	100	0
	*An. tessellatus*	4	100	0	0	100	0
	*An. aconitus*	3	30	0	70	33.3	66.7
	*An. minimus*	2	100	0	0	100	0
	*An. kochi*	1	100	0	0	100	0
	*An. annularis*	1	100	0	0	100	0
	*An. barbirostris*	1	100	0	0	100	0
Purworejo	*An. aconitus*	6	100	0	0	100	0
	*An. barbirostris*	4	100	0	0	100	0
	*An. vagus*	7	100	0	0	100	0
	*An. flavirostris*	3	100	0	0	100	0
	*An. annularis*	1	100	0	0	100	0
	*An. balabanceensis*	1	100	0	0	100	0
	*An. tessellatus*	1	100	0	0	100	0
	*An. maculatus*	1	100	0	0	100	0
Halmahera	*An. vagus*	5	100	0	0	100	0
	*An. kochi*	2	100	0	0	100	0
	*An. punctulatus*	1	100	0	0	100	0
	*An. barbumrosus*	1	100	0	0	100	0
	*An. farauti*	2	100	0	0	100	0

## Discussion

The molecular analysis of the *VGSC *gene of mosquitoes collected in three malaria endemic areas of Indonesia: South Lampung, Purworejo and South Halmahera, indicate the existence of the 1014F allele in four species of malaria vectors in Lampung: *An. sundaicus, An. subpictus*, *An. vagus *and *An. aconitus*. The 1014F and 1014S alleles were first reported among *Anopheles gambiae *in West Africa and East Africa respectively, and was later also reported in *Anopheles funestus, Anopheles albimanus, Anopheles sacharovi, Anopheles stephensi, Anopheles annularis, An. subpictus*, *Anopheles culicifacies *and *Anopheles vagus *from several places in Asia [13,15,17-19,29].

This is the first report of the existence of this resistance allele among *Anopheles *species in Indonesia and, therefore, has important implications for the management of malaria vector control in particular and integrated vector control in general. *Anopheles sundaicus *and *Anopheles subpictus *are major vectors of malaria in many coastal areas throughout western parts of Indonesia whereas *Anopheles aconicus *is a major vector in lowland areas [9,10]. *Anopheles vagus *is abundant across a wide variety of habitats in Indonesia including coastal, lowland and hilly villages. A recent report incriminated this species as potential malaria vector in Timor Leste [30].

The results also support a previous report of resistance to DDT in *An. sundaicus *from the Lampung area, and that the existence of this *kdr *allele may be associated with the extensive use of DDT over three decades [see review [21]]. Although DDT was banned in Indonesia in 1970, the use of pyrethroids in both IRS and mosquito nets for vector control and to a lesser extent, for agricultural use may also maintain the insecticide pressure over the mosquito population. In this context, it is interesting to note that resistance alleles were not found in Anopheline catches from Purworejo, Central Java, particularly *An. aconitus*, where resistance to DDT and pyrethroids have been previously documented [21]. This phenomenon may be associated with the fact that insecticide use in Java, particularly for malaria control has been significantly reduced following the success of the malaria control programme to bring malaria incidence down to hypoendemic status in certain foci [20]. However, as the *Anopheles *samples analysed were relatively few, it is important to extend the analyses to include more *Anopheles *samples and species from the surrounding area.

The findings also indicate the importance of insecticide susceptibility monitoring before introducing new insecticides to a particular area as well as regular insecticide resistance surveillance after the onset of a control programme. Currently, there are four classes of insecticides that are available in Indonesia: organochlorines, organophosphate, *Gamma aminobutyric acid *(GABA) inhibitors and pyrethroids that target different biochemical pathways in mosquito and other insects in general. Resistance to the aforementioned insecticides class in Indonesia has been reported in *Anopheles spp., Culex spp., Aedes spp.*, and other agricultural pests in various areas [21,31,32]. Sustainable insecticide resistance monitoring using bioassay and molecular tools would be an invaluable tool for providing information to decision makers at the Ministry of Health and Ministry of Agriculture to establish an integrated vector and pest control using appropriate insecticides.

## Conclusion

This study provides the first report of the existence of the insecticide-resistant allele, 1014F, in malaria vectors in Sumatera. It is important to explore the extent of the distribution of resistance alleles among the mosquito populations as various mosquito borne diseases such such as Dengue, Filariasis, Japanese Encephalitis and Chikungunya, are endemic to Indonesia

## Competing interests

The authors declare that they have no competing interests.

## Authors' contributions

DS, APNH, and PBSA performed molecular assays, data analysis, and the manuscripts writing. SS collected field samples and validate the morphological identification of the mosquito. DS, SS, NFL, and WAH design the study and manuscripts writing, and were responsible for management and fund raising for this study. All authors read and approved the manuscript.
